# Logic and memory functions of an inverter comprising reconfigurable double gated feedback field effect transistors

**DOI:** 10.1038/s41598-022-16796-x

**Published:** 2022-07-22

**Authors:** Juhee Jeon, Sola Woo, Kyoungah Cho, Sangsig Kim

**Affiliations:** Department of Electrical Engineering, Korea University, 145 Anam-ro, Seongbuk-gu, Seoul, 02841 Republic of Korea

**Keywords:** Electrical and electronic engineering, Nanoscale devices

## Abstract

In this study, we propose an inverter consisting of reconfigurable double-gated (DG) feedback field-effect transistors (FBFETs) and examine its logic and memory operations through a mixed-mode technology computer-aided design simulation. The DG FBFETs can be reconfigured to n- or p-channel modes, and these modes exhibit an on/off current ratio of ~ 10^12^ and a subthreshold swing (SS) of ~ 0.4 mV/dec. Our study suggests the solution to the output voltage loss, a common problem in FBFET-based inverters; the proposed inverter exhibits the same output logic voltage as the supply voltage in gigahertz frequencies by applying a reset operation between the logic operations. The inverter retains the output logic ‘1’ and ‘0’ states for ~ 21 s without the supply voltage. The proposed inverter demonstrates the promising potential for logic-in-memory application.

## Introduction

Recently, researchers have researched logic-in-memory (LIM) architectures using reconfigurable field-effect transistors (RFETs) combined with ferroelectric materials ^1,2^. The use of ferroelectric materials has allowed RFETs to possess memory characteristics while maintaining reconfigure behavior which switches n- and p-channel operation modes at transistor level^3–6^. As the reconfigurable behavior reduces the number of component transistors required for logic functionality in LIM architecture^7–10^, it enhances the logic functionality per circuit and enables various circuit topologies of LIM architecture^1^.

More recently, single-gated (SG) feedback field-effect transistors (FBFETs) operating in positive feedback mechanism have been researched to develop LIM architecture^11^. SG FBFETs have exhibited extremely low SS^12,13^, and the positive feedback mechanism enables SG FBFETs to perform logic and memory operations in LIM architecture comprising these transistors^11,14^. However, SG FBFETs have not exhibited desired reconfigurable behavior. Thus, in this study, we propose reconfigurable double-gated (DG) FBFETs and LIM operation of an inverter comprising these transistors. The DG FBFETs can be fabricated by CMOS-compatible top-down technology^19^. To demonstrate the LIM operation, transient simulations are performed through mixed-mode technology computer-aided design (TCAD) simulation. Moreover, by applying a reset operation, the inverter exhibits the LIM operation without the output voltage loss, a common problem in the FBFET-based inverters^12,15^.

### Simulation method

Our simulation was carried out with a two-dimensional structure using a commercial device simulator, Synopsys Sentaurus (O_2018.06)^16^. A DG FBFET had a p-i-n silicon nanowire (SiNW) structure with two gate electrodes (for details, see supplementary information). We used the thin layer mobility, the Lombardi, Philips unified mobility, and the high-field saturation model for considering the doping and field dependences of carrier mobility. Fermi statistics was applied to perform an accurate simulation. We also considered bandgap narrowing (default model), Shockley–Read–Hall (SRH) recombination with concentration-dependent lifetimes, Surface SRH recombination, and Auger recombination. Furthermore, the area factor of 12 nm was specified in the simulation for assuming the width of the transistor. In this study, we excluded the band-to-band-tunneling (BTBT) model because the BTBT is negligible in the DG FBFETs (for details, see supplementary information). In addition, the quantum confinement model was not considered because the dimension of the device is larger than the Fermi wavelength^20^.

## Results and discussion

### Switching and holding mechanisms of reconfigurable double-gated feedback field-effect transistors

**Figure 1:**
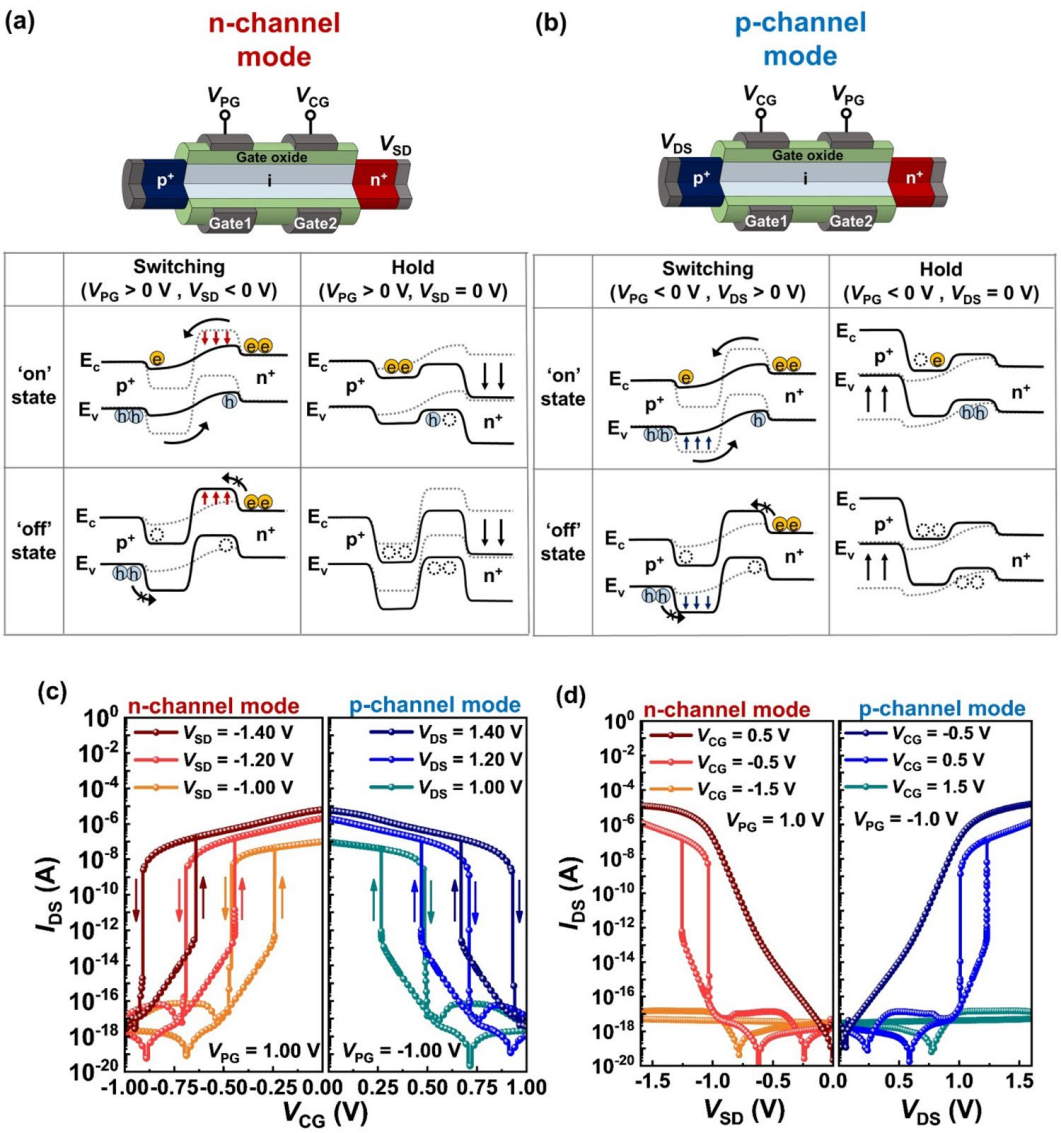
The operation mechanism of the reconfigurable double-gated feedback field-effect transistor (DG FBFET). The schematic illustration of the reconfigurable switching and holding operations in the (**a**) n- and (**b**) p-channel modes. (**c**) Transfer and (**d**) output characteristics for the n- and p-channel modes.

Figures 1a and b show the schematics band diagrams of the reconfigurable DG FBFETs during the switching and hold operations in the n- and p-channel modes. In the n-channel mode (Fig. 1a), gate1 operates as the program gate, whereas gate2 operates as the control gate. The positive bias of the program gate induces the virtual n-doped region in the intrinsic channel to block the hole injection from the drain. When a control-gate voltage (*V*_CG_) is positively swept with the negative source-to-drain voltage (*V*_SD_), electrons in the source are injected into the channel owing to the lowering of the potential barrier in the control-gated channel. The accumulation of electrons lowers the potential barrier in the program-gated channel, allowing the hole injection. As the potential barrier suddenly decreases due to the positive feedback loop, the transistor switches to the ‘on’ state with the latch-up phenomenon. However, when *V*_CG_ is negatively swept, the potential barrier suddenly rises with the elimination of the positive feedback loop. As a result, the transistor switches to the ‘off’ state with the latch-down phenomenon. During the ‘on’ state, excess charge carriers are accumulated in the potential well in the channel. When *V*_SD_ and *V*_CG_ are varied to 0 V, some holes release from the control-gated channel, but electrons remain in the program-gated channel because of the constant positive *V*_PG_ bias. Thus, the potential barrier in the program-gated channel is low enough to trigger the positive feedback loop. During the ‘off’ state, excess charge carriers are removed from the potential well in the channel. Thus, the potential barrier of the channel region is high enough to prevent the positive feedback loop at *V*_SD_ = *V*_CG_ = 0 V. In the p-channel mode (Fig. 1b), the position and polarity of the program- and control-gate are reversed. The negative *V*_PG_ (gate2) induces the virtual p-doped region that impedes the electron injection from the source. When a *V*_CG_ is negatively swept with the positive drain-to-source voltage (*V*_DS_), holes in the drain are injected into the channel, and then the transistor switches to the ‘on’ state with the latch-up phenomenon. Conversely, a positively swept *V*_CG_ leads to the switching ‘off’ operation with the latch-down phenomenon. When *V*_SD_ and *V*_CG_ are varied to 0 V in the ‘on’ state, the stored excess holes remain in the program-gated channel, lowering the potential barrier height enough to trigger the positive feedback loop. By contrast, for the ‘off’ state, the absence of excess charge carriers leads to a high potential barrier enough to prevent the positive feedback loop at *V*_SD_ = *V*_CG_ = 0 V.

Figure 1c shows the transfer characteristics of the reconfigurable DG FBFETs in the n- and p-channel modes. In the n-channel mode (*V*_PG_ = 1.00 V), as a *V*_SD_ varies from −1.00 to −1.40 V, the ‘switching on’ voltage (*V*_on_) shifts from −0.24 to −0.64 V. Contrary, in the p-channel mode (*V*_PG_ = −1.00 V), as a *V*_DS_ varies from 1.00 to 1.40 V, *V*_on_ shifts from 0.27 to 0.67 V. The shift of *V*_on_ is owing to the decrease in the potential barrier height between the drain (or source) and program-gated region. Moreover, for both channel modes, the transistor exhibits a constant memory window of ~ 0.25 V regardless of *V*_DS_ (or *V*_SD_). The transistor also exhibits an on/off current ratio of ~ 10^12 ^and a subthreshold swing (SS) of ~ 0.4 mV/dec at a *V*_DS_ (|*V*_SD_|) of 1.00 V for the p-channel (n-channel) mode. The output characteristics in the n- and p-channel modes are shown in Fig. 1d. When a *V*_CG_ of 0.5 V for the n-channel mode and -0.5 V for the p-channel mode is applied, the output characteristics are similar to the current–voltage characteristics of a p-n diode. However, when a *V*_CG_ of −0.5 V for the n-channel mode and 0.5 V for the p-channel mode are applied, the transistor exhibits the latch-up and -down phenomena. Moreover, the transistor is in the ‘off’ state when *V*_CG_ is −1.5 V for the n-channel mode and 1.5 V for the p-channel mode.

### Reset operation in the logic inverter comprising reconfigurable DG FBFETs

**Figure 2:**
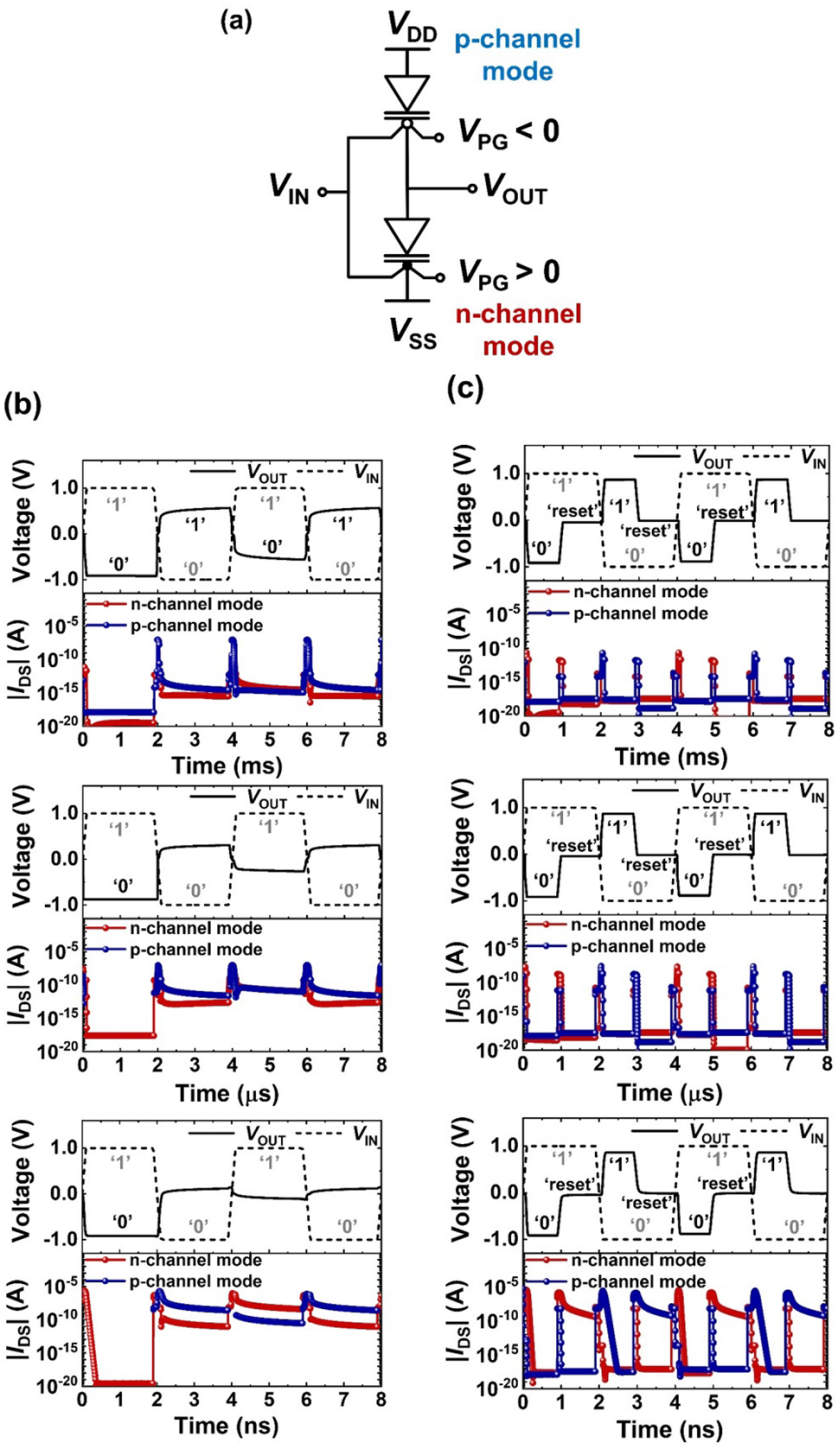
(**a**) Schematic of the inverter comprising the reconfigurable DG FBFETs. (**b**) Timing diagrams of the non-full swing inverting logic operation with ms, μs, and ns time steps under static conditions (a *V*_DD_ of 1.0 V, a *V*_SS_ of −1.0 V, and a |*V*_PG_| of 1.0 V). (**c**) Timing diagrams of the full swing inverting logic and reset operations with ms, μs, and ns time steps. The reset operation between the logic operations is carried out to eliminate the accumulated charge carriers; the reset operation takes place with *V*_IN_ = 1.0 V and *V*_PG_ = *V*_DD_ = *V*_SS_ = 0.0 V for the output logic ‘0’, and *V*_IN_ =  −1.0 V and *V*_PG_ = *V*_DD_ = *V*_SS_ = 0.0 V for the output logic ‘1’.

Figure 2a shows a circuit of the inverter comprising the reconfigurable DG FBFETs. In the proposed inverter, the top transistor operates in the p-channel mode for the pull-up operation, whereas the bottom transistor operates in the n-channel mode for the pull-down operation. The inverter is biased with supply voltages *V*_DD_ and *V*_SS_ corresponding to the drain voltage of the DG FBFETs in the p-channel mode and the source voltage of the DG FBFETs in the n-channel mode, respectively. The calculated output parasitic capacitance is 18 aF.

The transient responses of the inverting logic operation with ms, μs, and ns time steps are depicted in Fig. 2b. Under static voltage conditions (a *V*_DD_ of 1.0 V, a *V*_SS_ of −1.0 V, and a |*V*_PG_| of 1.0 V), input voltages (*V*_IN_) of 1.0 and −1.0 V are applied for the input logic ‘1’ and ‘0’, respectively. The inverter exhibits a *V*_SS_ level at the first output logic ‘0’ state, but an output voltage loss for the subsequent logic ‘1’. As the time step decreases from milliseconds to nanoseconds, the voltage level of the subsequent logic ‘1’ decreases from 0.6 to 0.1 V. Thus, the output voltage loss increases as the operating frequency increases. This non-full swing characteristic is attributed to the ‘data-retaining’ behavior that stores charge carriers in the channel of the DG FBFETs. During the pull-down operation at the first input logic ‘1’, charge carriers are accumulated and lower the potential barrier of the DG FBFETs in the n-channel mode. The faster the logic transition speed is, the more difficult it is to remove the accumulated charge carriers. Accordingly, at the subsequent input logic ‘0’, the off-current level of the DG FBFETs in the n-channel mode increases from ~ 10^–15^ to ~ 10^–11^ A as the time step decreases from milliseconds to nanoseconds. The situation is similar to the subsequent input logic ‘1’. However, the output voltage is settled at an intermediate voltage under the millisecond speed since it is hard to remove the accumulated charge carriers even at low frequencies. Hence, in order to resolve the output voltage loss, we use the reset pulse that plays a role in eliminating the accumulated charge carriers between the logic operations.

Figure 2c shows the transient responses of the inverting logic and reset operations with ms, μs, and ns time steps. The reset operation takes place with *V*_IN_ = 1.0 V and *V*_PG_ = *V*_DD_ = *V*_SS_ = 0.0 V for the output logic ‘0’ state, and *V*_IN_ = −1.0 V and *V*_PG_ = *V*_DD_ = *V*_SS_ = 0.0 V for the output logic ‘1’ state. During the reset operation, applying a *V*_PG_ of 0 V causes the emission of the residual charge carriers. Also, the removed supply voltage prevents unintended charge carrier injection, and an electric field formed in the channel region by *V*_IN_ removes the residual charge carriers. This charge carrier emission results in an off-current level of 10^–10^ A in the n-channel (p-channel) transistor during the resetting the output logic ‘0’ (‘1’) state under the speed of nanosecond order. For the input logic ‘0’ (‘1’), the off-current level of the n-channel (p-channel) transistor is ~ 10^–18^ A after the reset operation, thereby the inverter exhibits a *V*_DD_ (*V*_SS_) level regardless of the operating frequency. Since the output voltage (*V*_OUT_) at the reset operation is distinctive with the output logic ‘1’ and ‘0’ states, the inverter exhibits three logic states of ‘1’, ‘0’, and ‘reset’.

**Figure 3:**
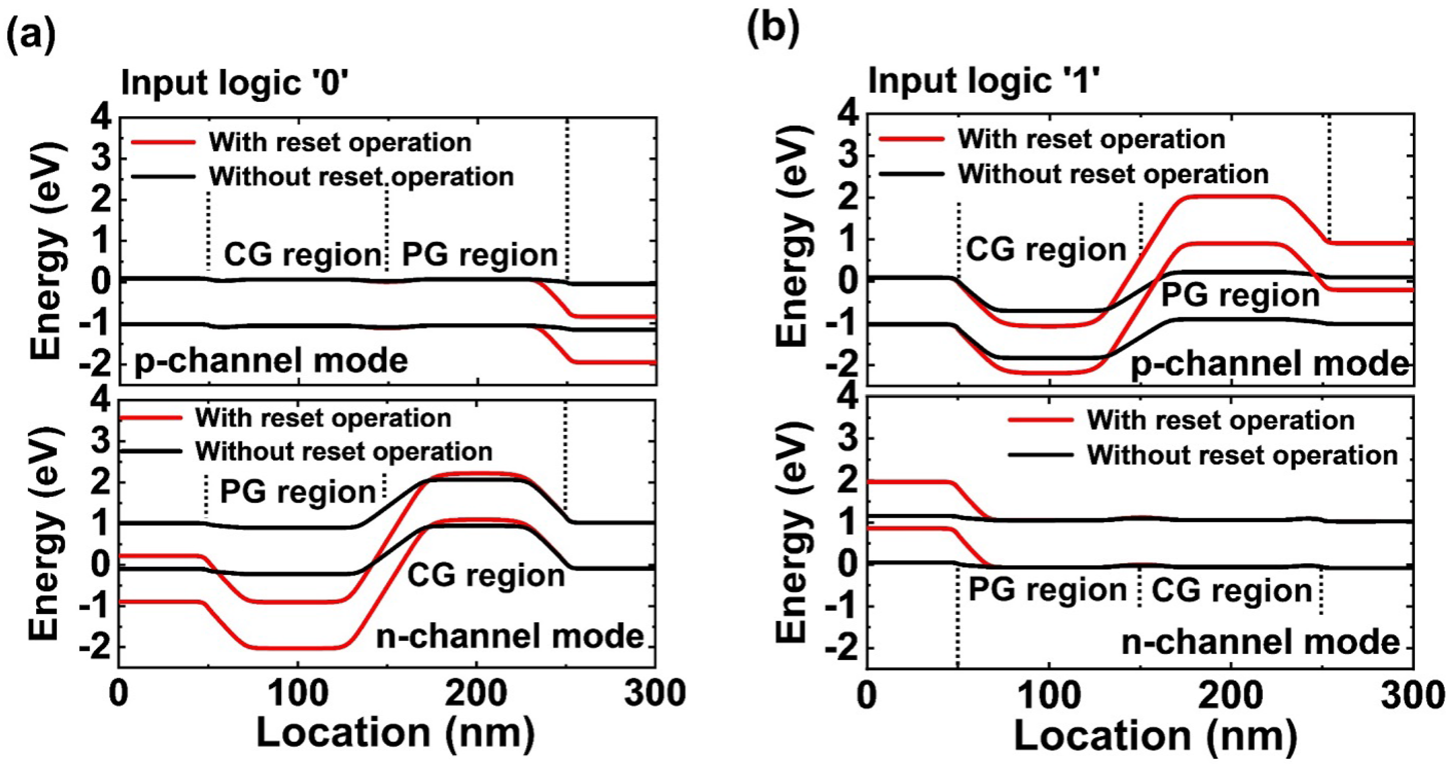
Energy band diagrams of the DG FBFETs in the n- and p-channel modes during the (**a**) input logic ‘0’ and (**b**) input logic ‘1’. The reset operation modulates the height of the potential barrier in the channel region. The data were extracted from the timing diagrams of the ns time step in Fig. 2(b) and (c).

To verify the effect of the reset operation, we analyze the energy band diagrams of the DG FBFETs in the p- and n-channel modes during logic operation under the speed of nanosecond order. For the input logic ‘0’ (Fig. 3a), the DG FBFETs in the p-channel mode is in the ‘on’ state while the DG FBFETs in the n-channel mode is in the ‘off’ state. In the DG FBFETs in the n-channel mode, the lowered potential barrier of the program-gated channel indicates that the residual electrons are mainly in the program-gated channel. Thus, resetting the residual electrons leads to the high potential barrier of the DG FBFETs in the n-channel mode enough to suppress the leakage current. Conversely, for the input logic ‘1’ (Fig. 3b), the DG FBFETs in the p-channel mode is in the ‘off’ state while the DG FBFETs in the n-channel mode is in the ‘on’ state. Without the reset operation, the presence of the residual holes lowers the potential barrier of the program-gated channel in the DG FBFETs in the p-channel mode. By resetting the residual holes, the DG FBFETs in the p-channel mode exhibits a high potential barrier even at the speed of nanosecond order.

### Logic and memory operation of the logic inverter

**Figure 4:**
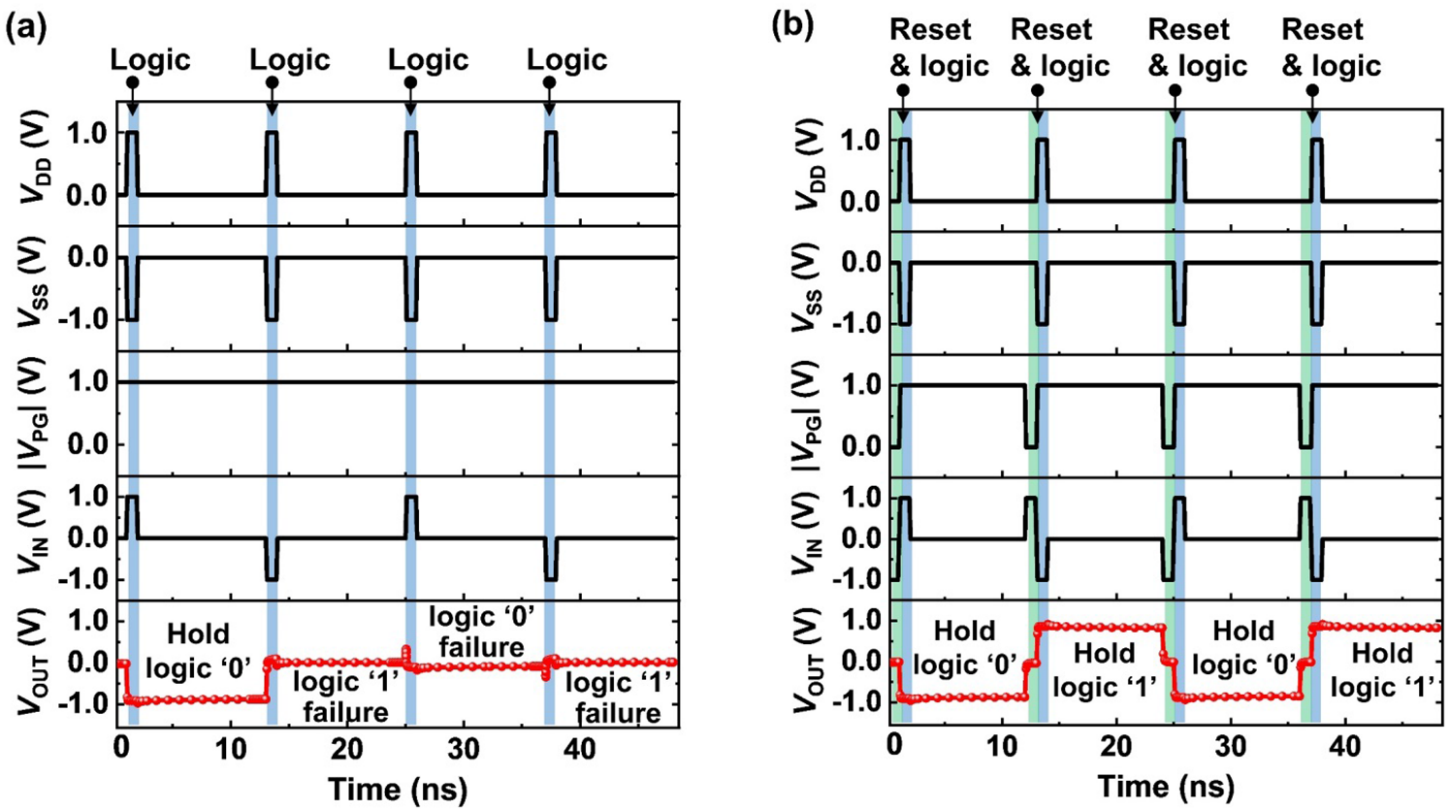
(**a**) Timing diagrams of consecutive logic and hold operations: hold logical data failure. (**b**) Timing diagrams of consecutive reset, logic and hold operations. The pulses of the drain, source, program-gate, input, and output voltages (*V*_DD_, *V*_SS_, *V*_PG_, *V*_IN_, and *V*_OUT_) are applied with a logic (reset) pulse width of 1 ns and a hold pulse width of 10 ns.

The presence of the residual charge carriers, which is an obstacle for logical calculation, enables the inverter to store its logical state without the supply voltage. Figure 4 shows the timing diagrams for analyzing the memory ability of the inverter. The blue and green columns indicate the input logic and reset operations, respectively, and both have a pulse width of 1 ns. For the hold operation, the biases of *V*_IN_ = *V*_DD_ = *V*_SS_ = 0.0 V and |*V*_PG_|= 1.0 V are applied for 10 ns. When input logic ‘1’ is applied at a *V*_DD_ of 1.0 V, a *V*_SS_ of -1.0 V, and a |*V*_PG_| of 1.0 V (Fig. 4a), the inverter exhibits a *V*_SS_ level as the output logic ‘0’ state. Further, when input and supply voltages are removed, *V*_OUT_ consistently retained a *V*_SS_ level as the output logic ‘0’ state without any voltage drops. However, when input logic ‘1’ is applied, the following output logic state is indistinguishable because of the output voltage loss. Therefore, the inverter needs a reset operation between the hold and logic operations to perform the memory function properly without failure. When a reset pulse is applied after the hold logic ‘0’ (Fig. 4(b)), the output logic transitions from ‘0’ to ‘reset’ state. Then, applying a *V*_IN_ of −1.0 V (with a *V*_DD_ of 1.0 V, a *V*_SS_ of -1.0 V, and a |*V*_PG_| of 1.0 V) changes the output logic state from ‘reset’ to ‘1’ for 1 ns without the output voltage loss. Further, the inverter exhibits a *V*_DD_ level as the output logic ‘1’ state during the hold operation. After the subsequent reset and input logic ‘1’ pulses, *V*_OUT_ retains a constant *V*_DD_ level for holding the output logic ‘1’ state.

**Figure 5:**
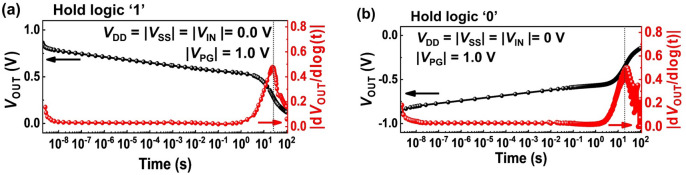
*V*_OUT_ and |d*V*_OUT_/dlog(t)| for holding the output logic (**a**) ‘1’ and (**b**) ‘0’ states. The time to reach maximum |d*V*_OUT_/dlog(t)| represents the retention time.

**Figure 6:**
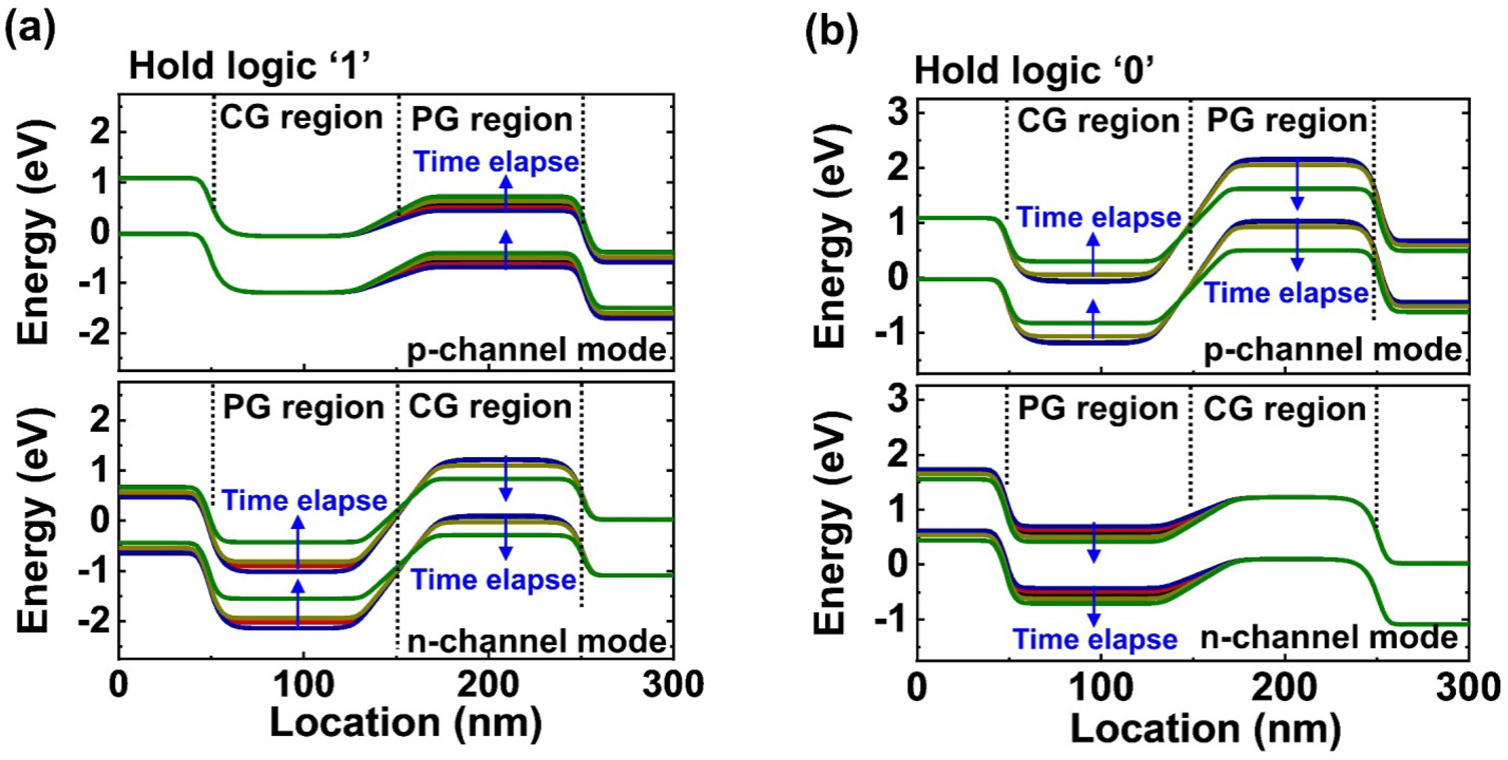
Energy band diagrams of the DG FBFETs in the n- and p-channel modes while holding the output logic (**a**) ‘1’ and (**b**) ‘0’ states.

Figure 5 shows the *V*_OUT_ during the holding operation as the semi-log plot. As time increases to 100 s, *V*_OUT_ of logic ‘1’ and ‘0’ states reaches 0 V. We consider the time to reach the maximum |d*V*_OUT_/dlog(t)|, i.e., inflection point, as the retention time of the stored logic states; The retention time of the logic ‘1’ and ‘0’ states is the same as ~ 21 s. For the logic ‘1’ (‘0’), a slope of *V*_OUT_ decrease (increase) is constant until ~ 1 s, and then it increases. The change of the slope is related to the potential barrier collapse of the ‘off’ state transistor. During holding logic ‘1’ (Fig. 6a), the stored holes leak out slowly from the program-gated region of the DG FBFETs in the p-channel mode until the high potential barrier of the DG FBFETs in the n-channel mode is maintained. However, owing to the continued charge carrier injection, the potential barrier of the DG FBFETs in the n-channel mode decreases rapidly after 1 s, and the *V*_OUT_ decrease accelerates. For the holding logic ‘0’ (Fig. 6b), the stored electrons leak out slowly from the DG FBFETs in the n-channel mode because of the high potential barrier the DG FBFETs in the p-channel mode is maintained. As the potential barrier of the DG FBFETs in the p-channel mode decreases rapidly after 1 s, the *V*_OUT_ increase accelerates.

We demonstrate that the proposed inverter can perform the logic operation and store the output. We also investigate the non-full swing characteristic that could be attributed to the ‘data-retaining’ behavior or the high latch-up/down voltage of the DG FBFETs. Moreover, by resetting the accumulated charge carriers, the inverter exhibits the *V*_DD_ or *V*_SS_ level at the output logic under gigahertz frequency. The equal voltage level between supply and logic signal facilitates the application of the inverter in a cascaded circuit. On the other hand, the reset operation with removing the supply voltages could be an issue when applying to an extended LIM system. Hence, further research is needed on the pulse schemes capable of resetting the accumulated charge carriers without removing the supply voltages to overcome the issue. Furthermore, the presence of the three distinct logic states of ‘1’, ‘0’, and ‘reset’ implies that the proposed inverter can be applied in ternary logic-in-memory systems^17,18^.

## Conclusion

In this study, we demonstrate the logic and memory functions of the inverter comprising the reconfigurable DG FBFETs. In the inverter based on the positive feedback mechanism, the output voltage loss arises from the residual charge carriers in the channel region during the logic operation. We apply the reset operation of removing the residual charge carriers with a program-gate, allowing the full pull-up and -down operations. As a result, the inverter exhibits the *V*_DD_ or *V*_SS_ level of the output logic state under a switching speed of nanoseconds order. Moreover, the output logic level of ‘1’ and ‘0’ is retained for ~ 21 s without the supply voltages. The results verify the possibility of application of the proposed inverter in LIM architecture.

## Supplementary Information

Supplementary Information.

## Data Availability

All data generated during this study are included in this published article (and its Supplementary Information files).
