# Supplementary material for: Logic and memory functions of an inverter comprising reconfigurable double gated feedback field effect transistors
Source: Sci Rep. 2022 Jul 22;12:12534. doi: 10.1038/s41598-022-16796-x (PMC9307848; doi:10.1038/s41598-022-16796-x)
Supplement: Supplementary file 1 — Supplementary Information. [file 41598_2022_16796_MOESM1_ESM.docx]

**Supplementary information**

**Logic and memory functions of an inverter comprising reconfigurable double gated feedback field effect transistors**

**Juhee Jeon, Sola Woo, Kyoungah Cho and Sangsig Kim^*^**

Department of Electrical Engineering, Korea University,
145 Anam-ro, Seongbuk-gu, Seoul 02841, Republic of Korea

* Corresponding author. Tel: +82-2-3290-3245; Fax: +82-2-3290-3894

E-mail address: sangsig@korea.ac.kr

**The dimensional parameters of the DG FBFETs.**

Figure S1 shows the schematic design of the reconfigurable double-gated feedback field-effect transistors (DG FBFETs). The two separated gate electrodes were arranged side-by-side on the intrinsic channel region with a gate length (*L*_G_) of 80 nm. We designed a metal work function of 4.0 eV for gate1 and 5.65 eV for gate2. The transistors have the dimensional parameters of a channel length (*L*_ch_) of 200 nm, a gap (*L*_gap_) of 10 nm, a thickness (*t*_si_) of 12 nm, and an Al_2_O_3_ gate oxide thickness (*t*_ox_) of 2 nm. The doping concentrations of the p-type drain, and n-type source regions were 1×10^19^ cm^−3^, and the intrinsic channel region was lightly n-type doped with a doping concentration of 1×10^16^ cm^−3^.


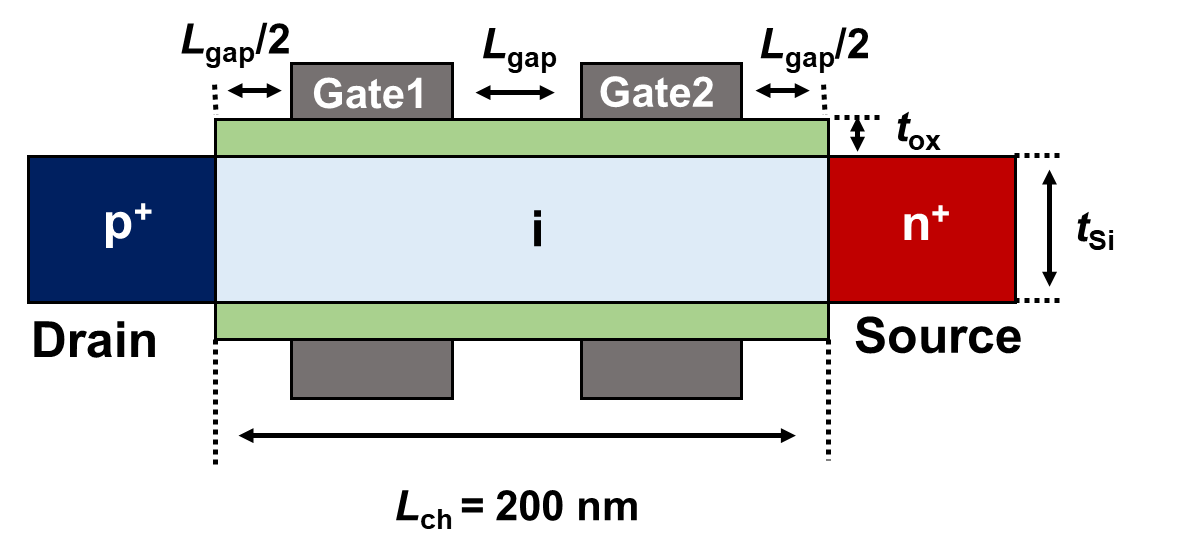


**Figure S1** Schematic design of the DG FBFETs

**The effect of band-to-band tunneling on the switching operation of the DG FBFETs.**

Even if the valance band edge of the control gate (CG) region overlaps with the conduction band edge of the program gate (PG) region (Fig. S2), the BTBT does not occur in the off-state because excess charge carriers are absent in the potential well of the intrinsic channel. Accordingly, we exclude the tunneling model in this study.


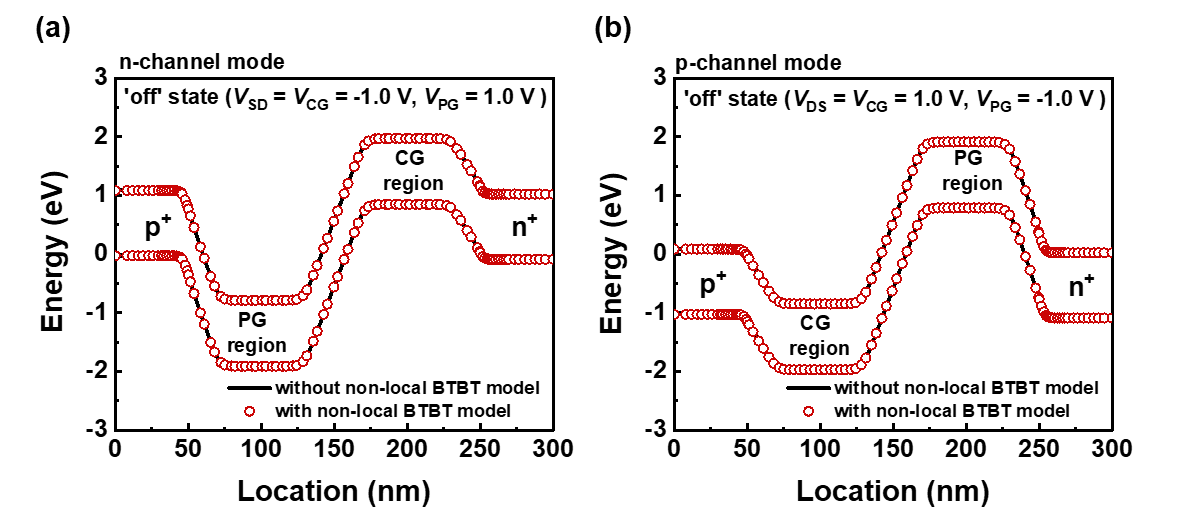


**Figure S2** Effect of the non-local BTBT model on the energy band diagram of the DG FBFETs in (a) the n-channel mode and (b) the p-channel mode in the ‘off’ state
